# Internal Ribosomal Entry Site-Mediated Translation Is Important for Rhythmic PERIOD1 Expression

**DOI:** 10.1371/journal.pone.0037936

**Published:** 2012-05-25

**Authors:** Kyung-Ha Lee, Sung-Hoon Kim, Do-Yeon Kim, Seunghwan Kim, Kyong-Tai Kim

**Affiliations:** 1 Division of Molecular and Life Science, Pohang University of Science and Technology, Pohang, Gyeongbuk, Republic of Korea; 2 School of Interdisciplinary Bioscience and Bioengineering, Pohang University of Science and Technology, Pohang, Gyeongbuk, Republic of Korea; 3 Department of Physics, Pohang University of Science and Technology, Pohang, Gyeongbuk, Republic of Korea; 4 Division of Integrative Biosciences and Biotechnology, Pohang University of Science and Technology, Pohang, Gyeongbuk, Republic of Korea; Pohang University of Science and Technology, Republic of Korea

## Abstract

The mouse PERIOD1 (mPER1) plays an important role in the maintenance of circadian rhythm. Translation of m*Per1* is directed by both a cap-dependent process and cap-independent translation mediated by an internal ribosomal entry site (IRES) in the 5′ untranslated region (UTR). Here, we compared m*Per1* IRES activity with other cellular IRESs. We also found critical region in m*Per1* 5′UTR for heterogeneous nuclear ribonucleoprotein Q (HNRNPQ) binding. Deletion of HNRNPQ binding region markedly decreased IRES activity and disrupted rhythmicity. A mathematical model also suggests that rhythmic IRES-dependent translation is a key process in mPER1 oscillation. The IRES-mediated translation of m*Per1* will help define the post-transcriptional regulation of the core clock genes.

## Introduction

A circadian rhythm, defined as an endogenously generated 24-hour-periodic oscillation, is found in most of living organisms from bacteria to human [1], [2]. Since all living things on the earth are influenced by the cycle of the sun, the robustness and the modulation of the self-sustained rhythm are important for efficiency of physiological processes and a quality of the life. The generation mechanism of the circadian rhythm has been mainly studied at the transcriptional and the post-translational level. Transcriptional activation of BMAL1/CLOCK heterodimer induces a synthesis of transcriptional repressors, such as *Period* (*Per*) [3], [4] and *Cryptochrome* (*Cry*) [5], [6] that have E-box motif at their promoter region, and PERIOD and CRYPTOCHROME protein form PER/CRY heterodimer at cytoplasm, then PER/CRY heterodimer translocates into the nucleus and represses BMAL1/CLOCK activation [5], [7]. In addition to the basic transcriptional feedback loop, several factors such as DEC1, 2 [8], [9], DBP [10], E4BP4 [11], [12] and NPAS2 [13], [14] are also identified as clock elements; moreover, a variety of kinases, phosphatase, acteylase, and ubiquitin ligases such as CK1δ/e [15]–[17], PP1 [18], [19], SIRT1 [20], [21], β-TRCP [22], and FBXL3 [23]–[25] are participated at the post-translational level. Combining all these factors, the circadian rhythm is able to sustain a 24-hour periodicity from the interlocked transcriptional and post-translational feedback loops.

Recent studies have been reported that post-transcriptional regulation is important for fine-tuning of the circadian rhythm. A few studies identified internal ribosomal entry site (IRES)-mediated translation modulated by RNA-binding proteins that play a role as IRES trans-acting factors (ITAFs) with binding to IRES-containing 5′-UTR of clock gene mRNA[26]–[28]. Several other studies showed mRNA degradation by RNA-binding proteins with their binding to 3′-UTR of clock gene mRNA[29]–[32]; therefore, these studies suggested that post-transcriptional regulation can modulate the amplitude and the phase of the circadian oscillation. Although relatively mild alteration might be derived by post-transcriptional regulation, it is important to understand how the rhythm is controlled in response to various external conditions.

*Period1* is one of the well-known clock genes in the mammalian circadian system. In accordance with the previous reports that *Per1* knockout mice show an altered period [33], the circadian expression of *Per1* is important in generation and maintenance of the rhythmicity. It was reported that rhythmic cap-independent translation mediated by HNRNPQ is taken place on the IRES in m*Per1* 5′UTR, and knock-down of HNRNPQ decreases the amplitude of PER1 protein oscillation without alteration of m*Per1* mRNA oscillation [28], suggested the evidence that post-transcriptional regulation is important for circadian m*Per1* expression. However, cellular IRES activity is typically lower than viral IRESs [34]. Indeed, the portion of IRES-mediated translation could be very low in overall translation of each gene [35].

Here, we compared IRES activity of m*Per1* with other genes. We present that m*Per1* IRES activity is critical to maintain the circadian rhythmicity of mPER1 protein through binding of HNRNPQ to specific region of m*Per1* 5′UTR. We also propose a mathematical modeling to explain molecular mechanisms of circadian rhythm-dependent m*Per1* translation.

## Results

### Cap-independent Translation of m*Per1*

**Figure 1:**
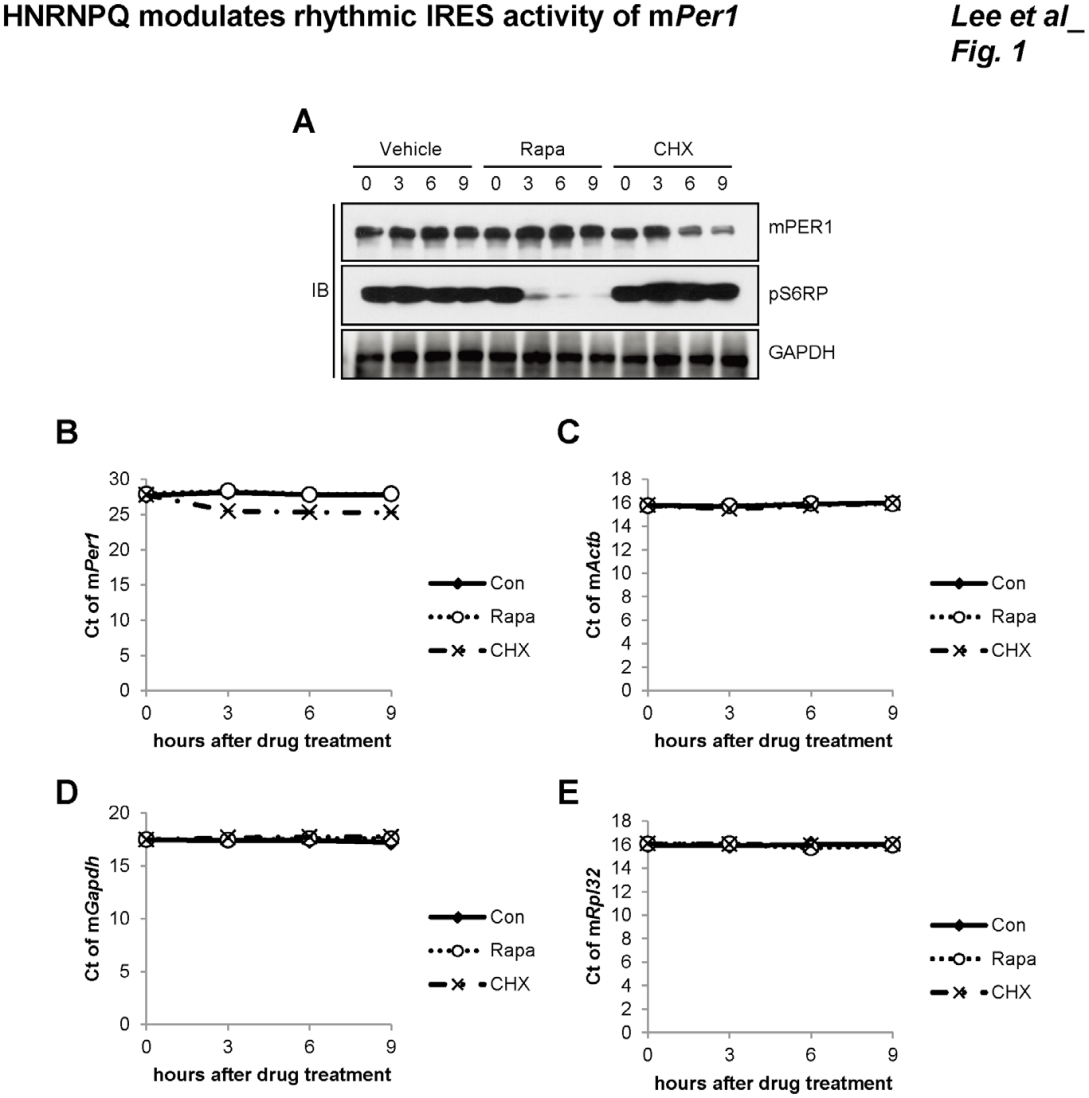
Cap-independent translation of m*Per1*. (A) Rapamycin (Rapa) or cycloheximide (CHX)-treated NIH 3T3 cells were harvested at indicated time points; then the protein levels were checked by immunoblotting. (B, C, D and E) Vehicle (DMSO)-, rapamycin (Rapa)-, or cycloheximide (CHX)-treated NIH 3T3 cells were harvested at the indicated time points; then mRNA levels were checked by real-time PCR with specific primers, (B) m*Per1*, (C) m*Actb*, (D) m*Gapdh* and (E) m*Rpl32*. mRNA levels were shown as cycle threshold (Ct) value.

Rapamycin induces hypophosphorylation of eIF4E-binding proteins (4E-BPs) and p70-S6 kinase (S6K1), causing inhibition of canonical cap-dependent translation [36], [37]. Phosphorylated active S6K1 can stimulate the initiation of protein synthesis through activation of S6 ribosomal protein (S6RP) and other components of the translational machinery [38]. When cells were treated with rapamycin to inhibit the cap-dependent translation, the levels of both phospho-S6 ribosomal protein (pS6RP) and phospho 4E-BPs were decreased, with no change in the level of mPER1 protein (Figure 1A and Figure S1). However, the general protein biosynthesis inhibitor, cycloheximide (CHX), induced a dramatic decrease in mPER1 protein. We also checked mRNA levels. Vehicle and cycloheximide did not change m*Per1* mRNA levels (Figure 1B). Rapamycin actually slightly increased m*Per1* mRNA levels. Nevertheless, rapamycin did not decrease mPER1 protein levels. Rapamycin and cycloheximide also did not change other housekeeping mRNA levels of mouse actin beta (m*Actb*), mouse glyceraldehyde-3-phosphate dehydrogenase (m*Gapdh*) and mouse ribosomal protein L32 (m*Rpl32*) (Figure 1C, D, E). We also checked real-time PCR results whether the PCR signals were in the linear range by showing amplification plot (Figure S2). These results suggest that an alternative translational system which is cap-independent translation can be involved in maintaining mPER1 protein levels.

### IRES Activity of m*Per1* 5′UTRs

**Figure 2:**
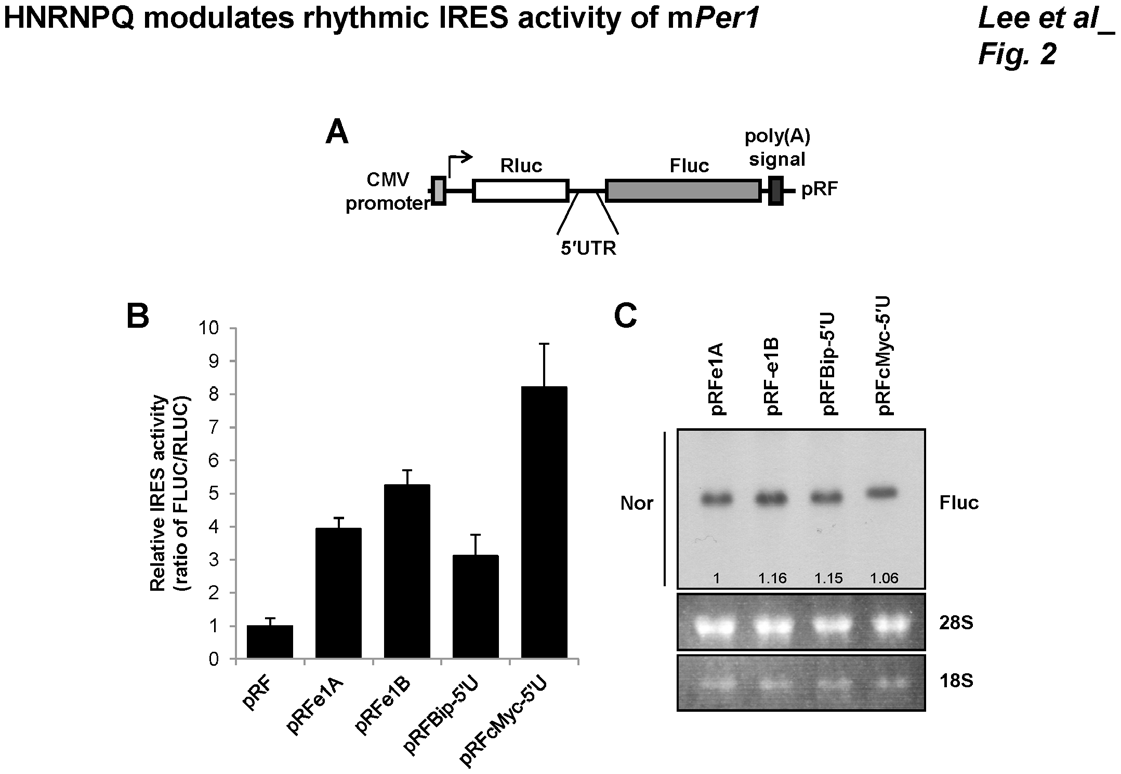
IRES activity of m*Per1* 5′UTRs. (A) Schematic diagram of bicistronic reporter plasmids. 5′UTRs were inserted into intergenic region between Rluc and Fluc. Bicistronic reporter plasmid (pRF), *Renilla* luciferase (Rluc), and *firefly* luciferase (Fluc). (B) NIH 3T3 cells were transiently transfected with bicistronic reporters that harbor 5′UTRs of *Per1*, *Bip*, and *c-Myc*. After 24 h incubation, cells were subjected to luciferase assay. The results are expressed as the mean ± SEM. (C) Bicistronic reporters that harbor 5′UTRs were transfected to HEK 293A cells. After 24 h, cells were harvested, and total RNAs were prepared and subjected to Northern blotting. Total RNA (2.5 µg) was hybridized with a specific probe for the *Fluc* coding region. 18S and 28S RNAs are shown as controls. The data was quantified by measuring the ratio of Fluc/28S.

m*Per1* has two forms of 5′UTRs (e1A:183 bp; e1B:194 bp) by alternative promoter usage. Two 5′UTRs are consisted of the first exon which is different from each other and the common second exon which has the start codon. Although the IRES activity of m*Per1* is reported previously, the extent of m*Per1* IRES activity was not known, and IRES activity of m*Per1* could be weak [28]. To know the strength of IRES activity of m*Per1*, we compared the IRES activity with other 5′UTRs which are well-known to have cellular IRES, heat shock 70 kDa protein 5 (HSPA5, also known as Bip) and v-myc myelocytomatosis viral oncogene homolog (c-Myc)[39]–[41] by using bicistronic reporter system. The bicistronic reporter plasmids produce bicistronic mRNA consisting of *Renilla* luciferase (*Rluc*), which is translated in a cap-dependent manner, followed by *Firefly* luciferase (*Fluc*) under the translational control of intergenic 5′UTR sequences (Figure 2A). FLUC activity reflects the IRES activity of the inserted intergenic sequences. The IRES activities of the m*Per1* 5′UTRs were stronger than those of the *Bip* 5′UTR and slightly weaker than those of the *c-Myc* 5′UTR (Figure 2B). The integrity of bicistronic mRNAs was also checked by Northern blotting, which confirmed that the induction of *Fluc* translation was not caused by altered mRNA stability, transcription, or the presence of cryptic promoter activity or splice acceptors that produce monocistronic products (Figure 2C). 5′UTRs of m*Per1* also did not change mRNA stability (Figure S3). These results suggest that IRES activity of m*Per1* is not weak but quite strong to modulate overall mPER1 protein levels.

### HNRNPQ Binding Site and m*Per1* IRES Activity

**Figure 3:**
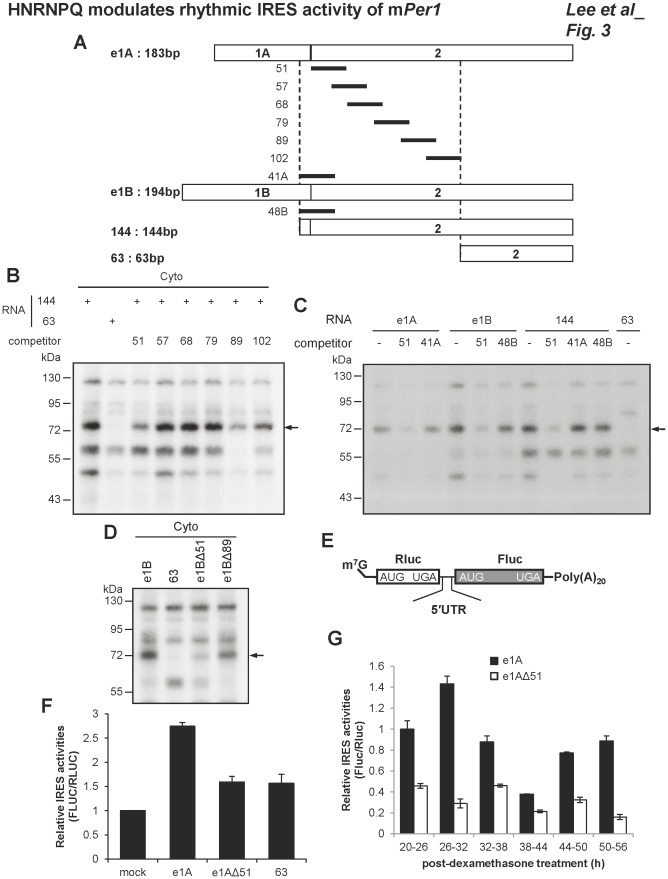
HNRNPQ binding site and m*Per1* IRES activity. (A) Schematic diagram of serially deleted mutation strategy and design of competitive oligonucleotides to perform UV cross-linking with oligonucleotide competition. (B and C) Radiolabeled *in vitro* transcribed RNAs were incubated with cytoplasmic extracts and competitive oligonucleotide for in vitro binding. Then UV cross-linking was performed. (D) Radiolabeled deletion mutants RNAs, e1BΔ51 and e1BΔ89, were subjected to UV cross-linking. (E) Schematic diagram of bicistronic mRNA reporter of m*Per1* 5′UTRs; 7-methyl-guanosine (m^7^G) and 20-nt-long poly(A) tail [poly(A)20]. (F) *In vitro* transcribed reporter mRNAs of 5′UTRs were transfected, then a luciferase assay was performed. The activity of the mock was set to 1 (n = 3). (G) Bicistronic mRNA reporters, e1A and e1AΔ51, were transfected into synchronized cells. After 6 h, cells were harvested at indicated time points; and then luciferase activity was checked. The activity of e1A at ∼20–26 time point was set to 1 (n = 3).

HNRNPQ was identified as an important ITAF for m*Per1* translation [28]. It was also reported that 144 m*Per1* 5′UTR reporter exhibited IRES activity similar extent to the full length 5′UTR of m*Per1*, but 63 reporter showed ∼70% decreased IRES activity compared to the full length. The truncated 63 reporter could not bind to HNRNPQ. The previous study concluded the region between 144 and 63 of the m*Per1* 5′UTR is important for IRES function (Figure 3A). Knockdown of HNRNPQ decreased immunoprecipitated HNRNPQ (Figure S4A). The samples immunoprecipitated by anti-HNRNPQ antibody in panel A were subjected to total RNA preparation, and m*Per1* mRNA levels were checked by real-time PCR. Knockdown of HNRNPQ dramatically reduced co-immunoprecipitated m*Per1* mRNA levels (Figure S4B). These results confirmed that the interaction between HNRNPQ and m*Per1* mRNA is specific. To identify important regions in the m*Per1* IRES for HNRNPQ binding more clearly, we designed and prepared oligonucleotides with specific sequences in the 5′UTR of m*Per1* (Figure 3A). The positions of competitive oligonucleotides were depicted as the asterisk on the top of nucleotides which are starting points of competitive oligonucleotides (Figure S5). UV cross-linking of HNRNPQ and m*Per1* 5′UTR was performed in the presence of competitive oligonucleotides. Competitive oligonucleotide 51 and 89 decreased the interaction between m*Per1* 5′UTR and HNRNPQ (Figure 3B). Although the sequence of oligonucleotide 51 partially overlaps sequences of competitor 41A and 48B, only 51 could compete with m*Per1* 5′UTR for HNRNPQ binding (Figure 3C). It is likely that both specific sequences in the m*Per1* 5′UTR and the secondary structure of the mRNA are important. From these results, we could narrow down the HNRNPQ binding region.

We deleted the competitor 51 or 89 region in the m*Per1* 5′UTR (e1AΔ51, e1AΔ89, e1BΔ51 and e1BΔ89) and UV cross-linking studies with these deletion mutant constructs revealed that the competitive oligonucleotide 51 region (e1AΔ51 and e1BΔ51) is important for HNRNPQ binding (Figure 3D). The IRES activities of m*Per1* 5′UTRs were monitored via transfection with bicistronic reporter mRNAs containing full length, e1AΔ51, and 63 of m*Per1* 5′UTR in the intercistronic regions (Figure 3E). The RNA transfection method was used to eliminate the possibility of aberrant mRNA production through a putative cryptic promoter or cryptic splicing acceptor in m*Per1* IRES that might be occurred when bicistronic mRNAs are generated by DNA transfection. When we transfected reporter mRNAs, IRES activity of the e1AΔ51 mutant was decreased similar to construct 63 reporter which does not have HNRNPQ binding region (Figure 3F). To verify the function of the m*Per1* IRES under physiological conditions with circadian rhythm, we transfected dexamethasone-treated synchronized cells with e1A or e1AΔ51 reporter mRNAs as a time course and then studied IRES activity. Wild-type m*Per1* 5′UTR e1A showed a rhythmic translation profile, but e1AΔ51 exhibited low IRES activity with dampened rhythmicity (Figure 3G). From these results we could find the HNRNPQ binding region in the m*Per1* 5′UTR, and demonstrate that HNRNPQ is important for rhythmic IRES activity.

### Rhythmic Phosphorylation of HNRNPQ

**Figure 4:**
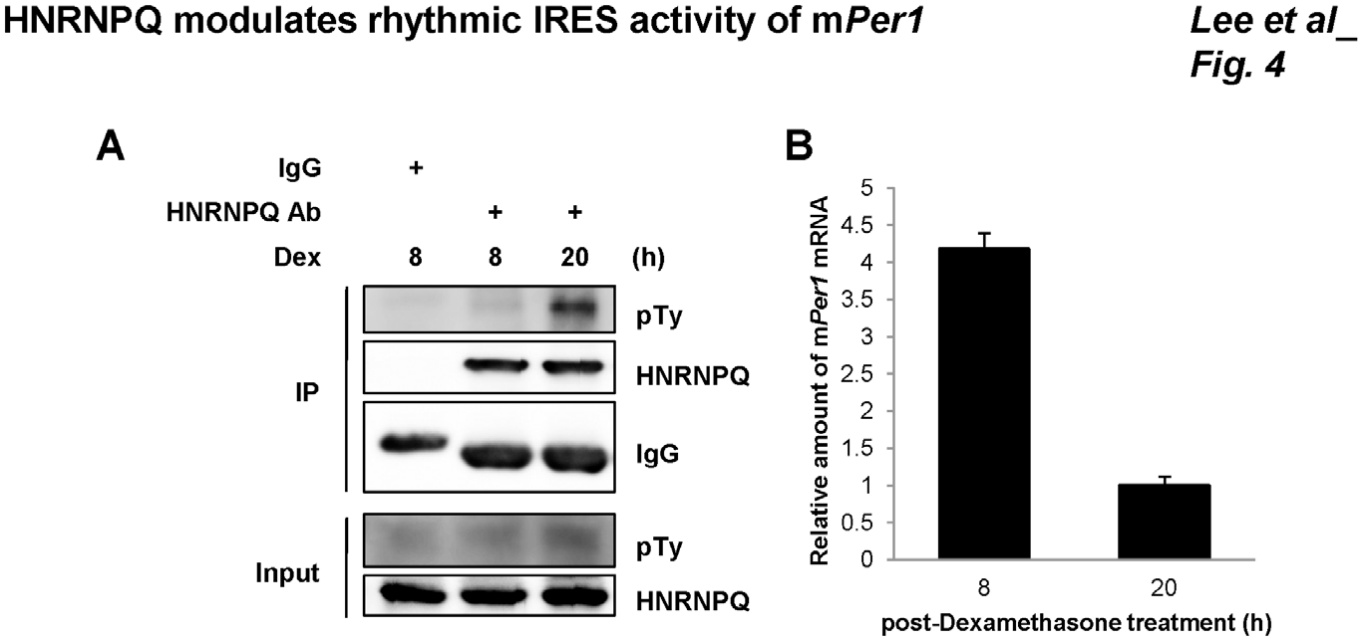
Rhythmic phosphorylation of HNRNPQ. (A) Dexamethasone-treated NIH 3T3 cells were harvested at the indicated time points, and then proteins were prepared under a phosphatase-free condition. Extracts were used for immunoprecipitation with HNRNPQ-specific antibody or IgG; then immonoblotting was performed with pTy- or HNRNPQ- specific antibodies. The blot for detection of HNRNPQ was stripped, and pTy bands were detected by pTy-specific antibody. (B) The one to fifth of the samples immunoprecipitated by HNRNPQ in panel A were subjected to total RNA preparation, then real-time PCR was performed.

HNRNPQ is important RNA binding protein for the translational regulation of *Nr1d1*, *Per1* and *Per3* [27], [28], [42]. Rather than the HNRNPQ protein itself exhibiting circadian rhythm, it was the interaction between HNRNPQ and mRNA that was rhythmic, and their binding was strongest at the protein peak time. Posttranslational modification of HNRNPQ, such as phosphorylation, may have an effect on the rhythmic interaction. HNRNPQ may be phosphorylated on tyrosine residue. It has been shown that the binding of RNA to HNRNPQ specifically inhibited HNRNPQ phosphorylation [43]. Based on this, we thought that phosphorylation of HNRNPQ might affect its binding affinity to mRNA. Therefore, we tested whether phosphorylation of HNRNPQ was time dependent; we found that tyrosine phosphorylation of HNRNPQ was rhythmic and showed a reciprocal profile to mPER1 (Figure 4A). We also confirmed by immunoprecipitation that the band detected with anti-pTy antibody was HNRNPQ. Co-immunoprecipitated m*Per1* mRNA by HNRNPQ antibody in Figure 4A showed higher level on 8 h than 20 h (Figure 4B). We assume that rhythmic phosphorylation of HNRNPQ may be one of the mechanisms allowing a time-dependent interaction between HNRNPQ and m*Per1* mRNA, and phosphorylated HNRNPQ may associate with m*Per1* mRNA more weakly.

### Mathematical Modeling

**Figure 5:**
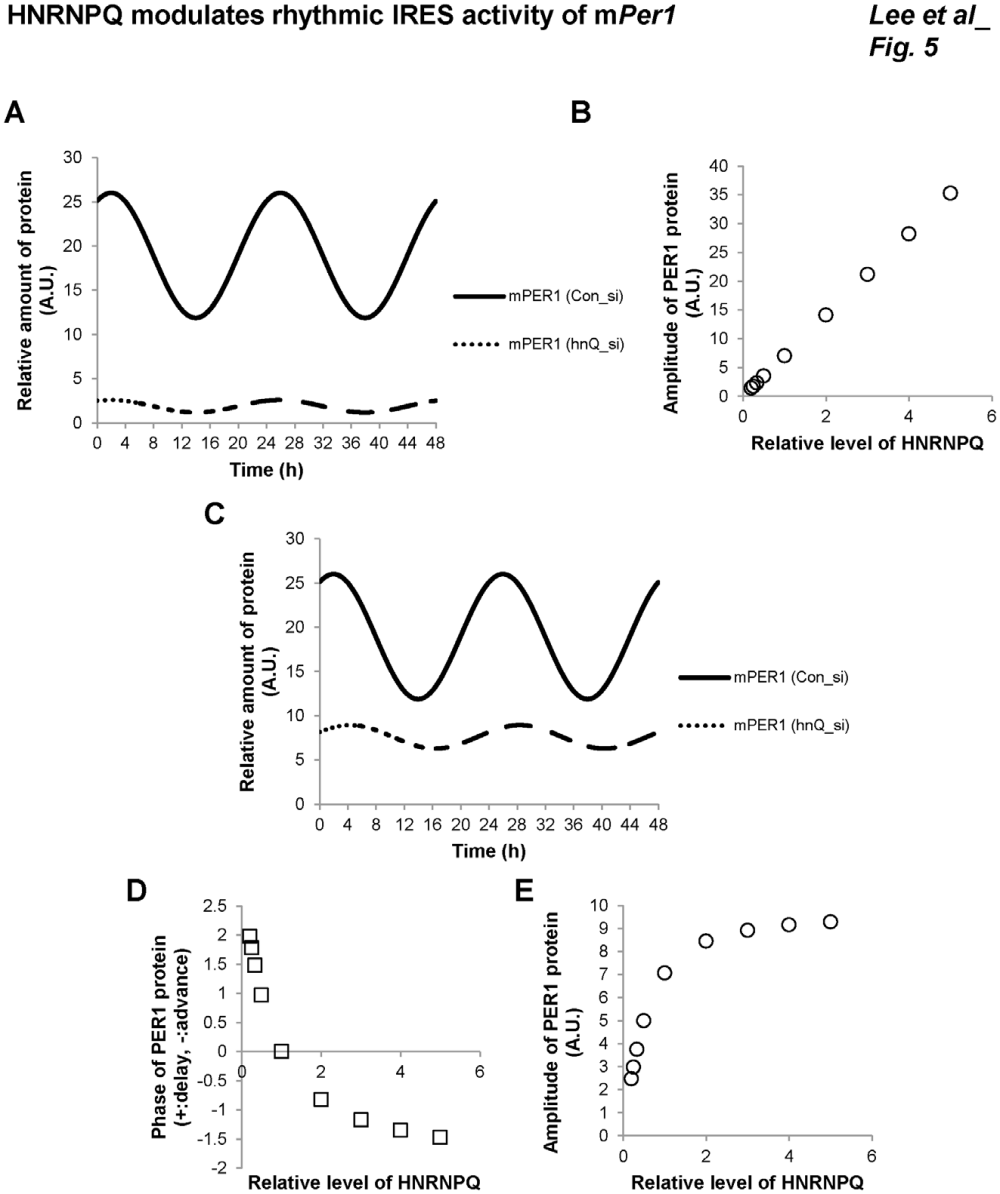
Mathematical modeling and summary. (A) Numerical simulation of the model describing the circadian PER1 protein expression. The solid and dotted curves indicate the level of mPER1 protein treated with Con_si and hnQ_si for HNRNPQ knockdown, respectively. (B) The relation between the amplitude of mPER1 protein and the level of HNRNPQ was obtained by numerical simulation using the model. (C) Numerical simulation of the model describing the circadian mPER1 protein expression with the assumption that mPER1 protein stability was influenced by the level of HNRNPQ. The solid and dotted curves indicate the level of mPER1 protein treated with Con_si and HNRNPQ-specific hnQ_si, respectively. (D) The model described mPER1 protein stability as a function of HNRNPQ and predicted the effect of HNRNPQ on both the amplitude and phase of the mPER1 protein oscillation. (E) The amplitude of mPER1 protein was described as a function of HNRNPQ levels. However, the relationship was not linear; mPER1 protein became saturated when HNRNPQ was abundant. (F) The proposed model for rhythmic translation of m*Per1* as a key regulatory mechanism of circadian mPER1 expression.

A mathematical model used in biology provides not only a theoretical background and systemic understanding of various biological phenomena but reasonable predictions without further experiments. To clarify the role of HNRNPQ in the IRES-mediated translation of m*Per1* mRNA in our study, a mathematical model was generated to describe our experimental results (see Methods). We fitted circadian m*Per1* mRNA and m*Per1* mRNA-bound HNRNPQ levels into the mathematical functions of the cosine with a period of 24 hours. The equation for mPER1 protein expression contained the processes of cap-dependent translation, cap-independent translation, and degradation. We assumed here that the m*Per1* mRNA-bound HNRNPQ level is proportional to the total HNRNPQ level, and HNRNPQ knockdown reduces both the m*Per1* mRNA-bound HNRNPQ level and the IRES-mediated translation of m*Per1* mRNA because the rate of IRES-mediated translation is proportional to the amount of HNRNPQ-bound m*Per1* mRNA. We also verified our assumption that knockdown of HNRNPQ decreases HNRNPQ associated m*Per1* mRNA (Figure S4A, S4B). Additionally, we assumed that mPER1 protein degradation is determined as the product of the coefficient for degradation rate and the amount of mPER1 protein. In our assumption, the coefficient for protein degradation was equal to ln2/t½ (mPER1 protein half-life), with t½ determined experimentally (data not shown). The simulation results showed the temporal variations in the amount of mPER1 protein depended on the HNRNPQ level. The results were consistent with the experimental data, showing that the amount of mPER1 protein was reduced as the HNRNPQ level decreased (Figure 5A); moreover, the model suggests that the amount of mPER1 protein is linearly proportional to the HNRNPQ level (Figure 5B). In such a condition, however, we could not obtain the phase delay of mPER1 protein according to the HNRNPQ knockdown. We introduced a term for mPER1 protein stability correlated with HNRNPQ level into the equation, since m*Per1* mRNA was not influenced by HNRNPQ.

We introduced into the equation a term for mPER1 protein stability as a function of HNRNPQ; the phase of mPER1 protein became delayed when the HNRNPQ level was decreased (mimcs a knockdown condition) (Figure 5C), and the phase of mPER1 protein became advanced when the HNRNPQ level was increased (mimics an over-expression condition) (Figure 5D). The amplitude of mPER1 protein was also influenced by the HNRNPQ level as expected, but the amplitude of mPER1 protein became saturated with excess level of HNRNPQ (Figure 5E). These results suggest that IRES-mediated translation of m*Per1* mRNA by HNRNPQ is important to determine the circadian oscillation of mPER1 protein.

## Discussion

m*Per1* is an important clock component that is part of the core feedback loop in the circadian rhythm system [3], [4]. m*Per1* is thought to be essential for maintaining biological rhythm and phase resetting [33], [44]. Recently, it was reported that expression of m*Per1* is mediated by IRES-dependent translation [28]. IRES activity of m*Per1* showed rhythmicity during circadian time and rhythmic expression of mPER1 was mediated by time dependent interaction between HNRNPQ and m*Per1* mRNA.

In the present study, we compared IRES activity of m*Per1* with other genes which are well-established cellular IRESs. From these results, we could find that the IRES activity of m*Per1* was quite potent that enough to modulate circadian rhythm. However, cellular IRES activity is typically lower than viral IRESs [34]. Indeed, translation rate constants of each cellular genes are variable and not uniform [45]. IRES activity of some cellular genes is weak, however it could be critical for the translation of those genes [46]–[48]. To know the potency of m*Per1* IRES more clearly, checking the portion of IRES-mediated translation in overall m*Per1* translation is needed.

We could also determine quite selective HNRNPQ binding region in the m*Per1* 5′UTR. Deletion of HNRNPQ binding region (e1AΔ51 construct) showed marked decrease in IRES activity with dampened rhythmicity. But HNRNPQ binding was not completely disappeared in e1AΔ51 (Figure 3D). We think that the deleted region of e1AΔ51 is important for HNRNPQ binding, but other region also contributes to the binding.

There were rhythmic changes in the level of phospho-HNRNPQ during circadian time (Figure 4). Our results suggest that differential phosphorylation of HNRNPQ during circadian time could occur and result differential binding of HNRNPQ to m*Per1* 5′UTR. HNRNPQ can be phosphorylated by several kinases, including protein kinase C [49], insulin receptor tyrosine kinase [43], and probably by ATM or ATR [50], [51]. Among them, only insulin receptor phosphorylated the tyrosine residue of HNRNPQ [43]. A few reports have suggested a role for tyrosine kinases in circadian regulatory mechanisms. In the mammalian suprachiasmatic nucleus, the Src-family tyrosine kinase Fyn proto-oncogene (*Fyn*) appears to be involved in the regulation of the circadian core oscillator, as *Fyn* ^−/−^ mutant mice shows a significantly longer circadian period than that of wild-type mice [52]. It has been shown that Src-family members, including c-Src, Lck and c-Yes, were expressed in the retina[53]–[56], and Src-family tyrosine kinases have been shown to be activated in the retina on photic stimulation [57]. At present, it is not clear which circadian regulated tyrosine kinases and phosphatases are involved in HNRNPQ phosphorylation. To further clarify the relationship between m*Per1* mRNA and HNRNPQ with overall circadian system, it would be valuable to find the protein kinase and phosphatase responsible for HNRNPQ phosphorylation.

The possibility that HNRNPQ modulates other clock genes also should be considered. The results indicated that HNRNPQ could directly bind to the 3′UTR of m*Cry1* (Figure S6). As HNRNPQ binds to mRNA of m*Per1* and other clock genes, the knockdown of HNRNPQ or m*Per1* can lead to a different outcome. To understand the function of HNRNPQ in the overall clock system, further studies of the core clock protein levels should be done.

We have defined the role of HNRNPQ in IRES-mediated mPER1 protein translation and interpreted the regulatory processes with a mathematical equation. From our observations, m*Per1* mRNA oscillated over a period of 24 h was not significantly influenced by HNRNPQ knockdown. In addition, with the level of HNRNPQ constant, the level of HNRNPQ-bound m*Per1* mRNA oscillated. To generate a mathematical model describing mPER1 protein expression as a function of HNRNPQ, we assumed that the synthesis and degradation of HNRNPQ determined the level of mPER1 protein and wrote the mathematical terms according to the law of mass action. HNRNPQ participates in cap-independent translation as an ITAF. Therefore, HNRNPQ knockdown influences cap-independent translation. With the additional assumption that protein degradation is proportional to the amount of mPER1, we numerically simulated the model and confirmed the role of HNRNPQ on mPER1 protein expression. This result was consistent with findings, showing that the amplitude of mPER1 protein was a function of the level of HNRNPQ, but it did not show the phase delay in mPER1 protein after HNRNPQ knockdown. When we introduced mPER1 protein degradation rate as a function of the amount of HNRNPQ, we were able to demonstrate the phase delay in mPER1 protein oscillation. The relationship between HNRNP Q and mPER1 protein needs to be explored in further studies.

## Material and Methods

### Cell Culture, and Drug Treatment

NIH 3T3 cells were obtained from Korean Cell Line Bank (KCLB No. 21658). NIH 3T3 cells were cultured in DMEM (HyClone) with 10% fetal bovine serum (HyClone) and 1% antibiotics (WelGENE) and maintained in a humidified 95% air/5% CO_2_ incubator. The circadian oscillation of NIH 3T3 cells was synchronized by treatment with 100 nM dexamethasone. After 2 h, the medium was replaced with complete medium [31], [32]. To block the translation system, NIH 3T3 cells were treated with 20 nM rapamycin or 100 µg/ml cycloheximide, and then harvested at the indicated times.

### Plasmid Constructions

m*Per1* 5′UTRs (e1A, e1B) were amplified from m*Per1* cDNA using Pfu polymerase (Solgent). The resulting products were cloned into the SalI/SmaI site of the intercistronic region of a pRF bicistronic vector containing *Renilla* luciferase (*Rluc*) in the first cistron and *firefly* luciferase (*Fluc*) in the second cistron [26].

For the in vitro binding assay/UV cross-linking, fragments of m*Per1* 5′UTR were amplified, and the PCR products were digested and subcloned into the EcoRI/XbaI site of the pSK′ vector [29] to generate pSK′-e1A, pSK′-e1B, pSK′-144, and pSK′-63. To perform UV cross-linking with oligonucleotides competition, pSK′-e1AΔ51, pSK′-e1AΔ89, pSK′-e1BΔ51 and pSK′-e1BΔ89 were generated.

To generate the bicistronic mRNA reporter for mRNA transfection, pCY2-RFe1A, pCY2-RFe1B, and pCY2-RF63 were constructed as follows: The 5′UTRs of m*Per1* were cut from pRFe1A and pRFe1B and pRF63 using SalI/BamHI and inserted into the SalI/BamHI site of pCY2-RF [26]. pCY2-RFe1AΔ51, pCY2-RFe1AΔ89, pCY2-RFe1BΔ51, and pCY2-RFe1BΔ89 were constructed by deletion mutagenesis with Dpn I digestion.

### Transient Transfection

For expression of the reporter constructs in NIH 3T3 cells, the Neon® Transfection System (Invitrogen) was used as recommended by the manufacturer. The reporter mRNA transfection was performed as follows: NIH 3T3 cells were transiently transfected with 2 µg of the capped bicistronic reporter mRNA using lipofectamine2000 (Invitrogen) and incubated for 6 h. In the case of time-dependent transfection, NIH 3T3 cells were treated with dexamethasone and transiently transfected with 2 µg of the capped bicistronic reporter mRNA at intervals and incubated for 6 h for harvest.

### *In vitro* RNA Synthesis, *in vitro* Binding, UV Cross-linking

For *in vitro* binding assays, [^32^P]UTP-labeled RNA was transcribed from XbaI-linearized recombinant pSK′ vectors with T7 RNA polymerase (Promega). For mRNA transfection, the bicistronic pCY2 plasmids were linearized with EcoRI. This plasmid contains a 20-nt-long poly(A) tract between XhoI and EcoRI restriction sites. Reporter mRNA was generated *in vitro* from the linearized plasmid with SP6 RNA polymerase (Promega) in the presence of the ribo m^7^G cap analog (Promega).

*In vitro* binding and UV cross-linking were performed as previously described [26]. Briefly, equal amount of labeled RNAs were incubated with 30 µg cytoplasmic extracts from NIH 3T3 cells for 20 min. After incubation, the samples were UV-irradiated on ice for 15 min with a CL-1000 UV-crosslinker (UVP). Unbound RNA was digested with 5 µl RNase cocktail (RNase A and RNase T_1_). The reaction mixtures were analyzed by SDS-PAGE and autoradiography. For UV cross-linking and oligonucleotides competition, oligonucleotides were added at 1 µM to the RNA-protein binding reaction mixtures and UV cross-linking was performed. The sequences of competitive oligonucleotides are provided in Table S1.

### RNA Quantification, Immunoprecipitation-RT-PCR

mRNA levels were detected by quantitative real-time PCR using StepOnePlus real-time PCR system (Applied Biosystems) as previously described [28]. Immunoprecipitation-RT-PCR was performed as previously reported [28]. In briefly, immunoprecipitation was performed under RNase-free condition. RNA was extracted from the one fifth volume of washed agarose bead with an RNA isolation solution (Molecular Research Center). Then, reverse transcription and quantitative real-time PCR were performed.

### Immunoblot Analysis

Immunoblot analyses were performed with polyclonal anti-PER1, monoclonal anti-HNRNPQ (SIGMA), polyclonal anti-phospho-S6 ribosomal protein (Ser 235/236; Cell signaling), monoclonal anti-GAPDH (Millipore), monoclonal PY-20 (Transduction Laboratories), polyclonal anti-phospho 4EBP (Cell Signaling) and monoclonal anti-14-3-3ζ (Santa Cruz Biotechnology) as primary antibodies. HRP-conjugated species-specific secondary antibodies (KPL) were visualized using a SUPEX ECL solution kit (Neuronex) and a LAS-4000 chemiluminescence detection system (FUJI FILM). Acquired images were analyzed using Image Gauge (FUJI FILM) according to the manufacturer’s instructions.

### Mathematical Modeling

Based on our observations, the total m*Per1* mRNA and HNRNPQ Q-bound m*Per1* mRNA curves were fitted into the cosine waves with a period of 24 h as
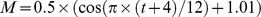
And



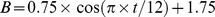
where M and B are the relative amounts of total m*Per1* mRNA and HNRNPQ -bound m*Per1* mRNA, respectively, and t is circadian time. Likewise, we described the level of HNRNPQ as constant and ineffective in m*Per1* mRNA oscillation. We assumed that the level of HNRNPQ does not influence the rate of cap-dependent translation but does influence the rate of cap-independent translation. Based on the law of mass action, the equation for the time derivative of mPER1 protein was generated from our assumptions that the rate of cap-dependent translation is directly proportional to the level of m*Per1* mRNA, the rate of cap-independent translation is proportional to the level of HNRNPQ-bound m*Per1* mRNA, and the rate of protein degradation is linearly proportional to its own level. Thus,



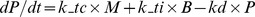
where M, B, and P are the relative amounts of total m*Per1* mRNA, HNRNPQ-bound m*Per1* mRNA, and mPER1 protein, respectively. The parameters k_tc, k_ti, and kd in the equation indicate the coefficients for cap-dependent translation, cap-independent translation, and protein degradation, respectively. The coefficient for protein degradation is equal to ln2/t½, where t½ is the protein half-life, and other parameters in the equation are chosen, as the numerically integrated protein curve is well-fitted into the experimental observation. The values of the parameters in our study are: k_tc  = 0.01, k_ti  = 5, and kd  = 0.462. The effect of HNRNPQ knockdown is shown in our equation as proportional to the rate of cap-independent translation. In other words, the rate of cap-independent translation is equal to the product of the relative level of m*Per1* mRNA-bound HNRNPQ and the basal rate of cap-independent translation.

## Supporting Information

Figure S1 **Cap-independent translation of m** ***Per1*** **.** NIH 3T3 cells were treated with vehicle (DMSO), rapamycin (Rapa), or cycloheximide (CHX), and cells were harvested at indicated time points. Harvested cells were subjected to immunoblotting with PER1, p4EBP, or GAPDH specific antibodies.
(TIFF)

Figure S2 **Amplification plots of real-time PCR.** Vehicle (DMSO)-, rapamycin (Rapa)-, or cycloheximide (CHX)-treated NIH 3T3 cells were harvested at the indicated time points; then mRNA levels were checked by quantitative RT-PCR with specific primers (Figure 1B–E). To indicate whether the PCR signals were in the linear range, amplification plots are shown. (A) m*Per1*, (B) m*Actb*, (C) m*Gapdh*, (D) m*Rpl32*.
(TIF)

Figure S3 **mRNA stability of m** ***Per1*** **5′UTRs.** NIH 3T3 cells were transiently transfected with monocistronic reporter plasmids that 5′UTR is followed by *Firefly* luciferase. Transfected cells were incubated for 24 h before treatment with 5 µg/ml actinomycin D. Total RNA (1 µg) was reverse transcribed using oligo-dT primer then quantified by real-time PCR. Closed square indicates mRNA levels of Fluc which harbor no 5′UTR. Open circle (e1A) and X (e1B) represent mRNA levels of Fluc which is linked to m*Per1* 5′UTR. The results are expressed as the mean ± SEM.
(TIF)

Figure S4 **Binding specificity between HNRNPQ and m** ***Per1*** **mRNA.** (A) Cytosolic fraction of NIH 3T3 transfected with Control siRNA (Con_si) or HNRNPQ specific siRNA (hnQ_si) were subjected to IP-RT using HNRNPQ specific antibody followed by immunoblotting. (B) Total RNA was prepared from the one fifth volume of the samples immunoprecipitated with anti-HNRNPQ antibody in panel A, and m*Per1* mRNA was detected by real-time PCR. The level of Con_si was set to 100. Error bars represent ±SEM.
(TIF)

Figure S5 **mRNA sequence of the m** ***Per1*** **5′UTR and the positions of competitive oligonucleotides.** 5′UTRs of m*Per1*, e1A and e1B, were presented. Blue colored sequence is the exon1 of e1A m*Per1* 5′UTR, green colored sequence indicates the exon1 of e1B. m*Per1* 5′UTRs e1A and e1B commonly have exon2, which was showed by red color. The starting points of competitive oligonucleotides were depicted as asterisk on the top of nucleotide.
(TIF)

Figure S6 **HNRNPQ specifically binds to the m** ***Cry1*** **3′UTR.** 3′UTRs of m*Cry1* transcribed *in vitro* were subjected to *in vitro* binding and UV cross-linking with a cytoplasmic extract. Cytoplasmic extracts labeled by UV cross-linking were subjected to immunoprecipitation with antibodies against HNRNPQ or pre-immune serum as a control.
(TIF)

Table S1 Sequences of competitive oligonucleotides.
(TIF)
