# Supplementary figures and images for: Internal Ribosomal Entry Site-Mediated Translation Is Important for Rhythmic PERIOD1 Expression
Source: PLoS One. 2012 May 25;7(5):e37936. doi: 10.1371/journal.pone.0037936 (PMC3360671; doi:10.1371/journal.pone.0037936)

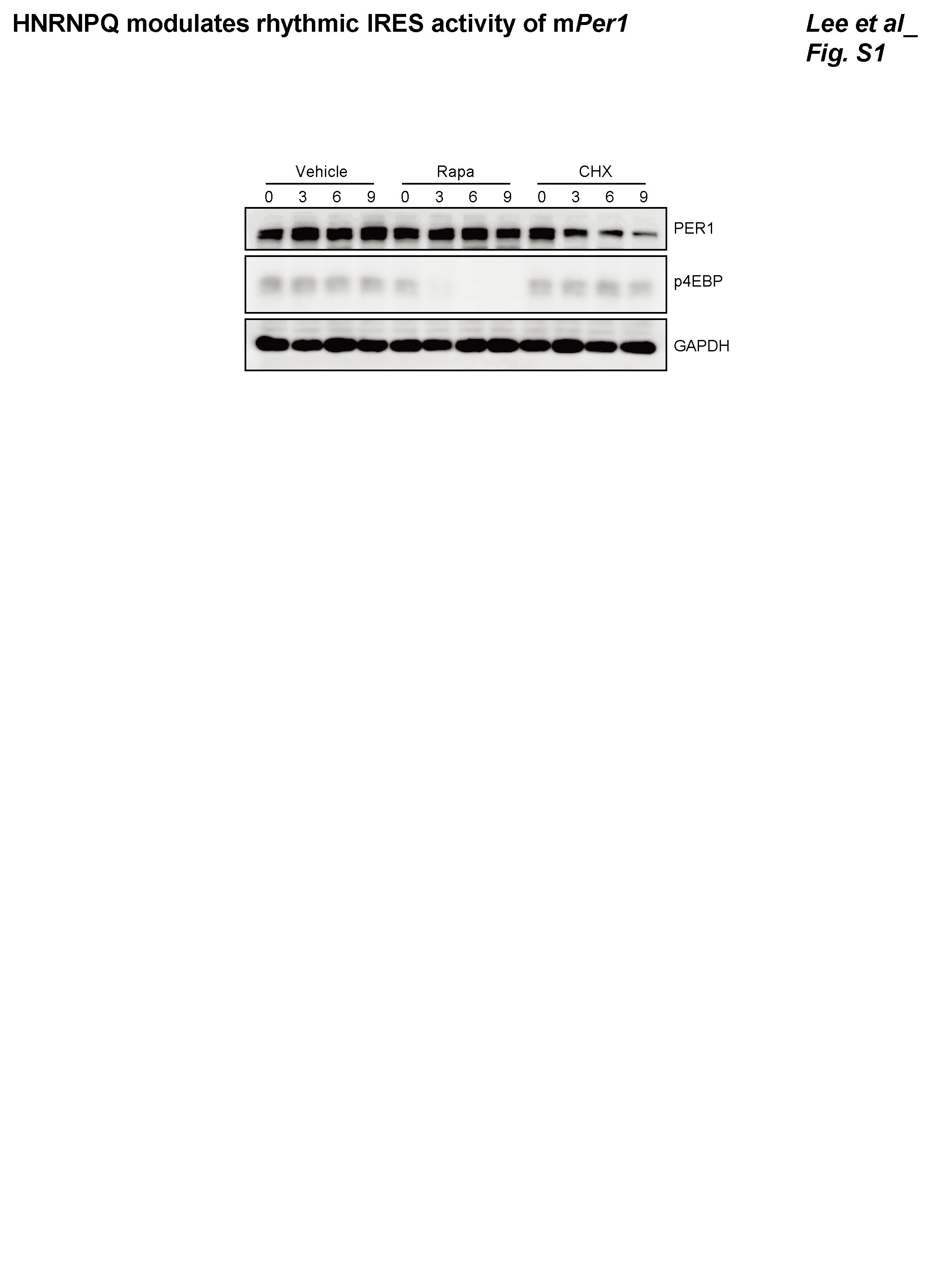

Supplement: Figure S1 — Cap-independent translation of m Per1 . NIH 3T3 cells were treated with vehicle (DMSO), rapamycin (Rapa), or cycloheximide (CHX), and cells were harvested at indicated time points. Harvested cells were subjected to immunoblotting with PER1, p4EBP, or GAPDH specific antibodies. (TIFF) [file pone.0037936.s001.tiff]

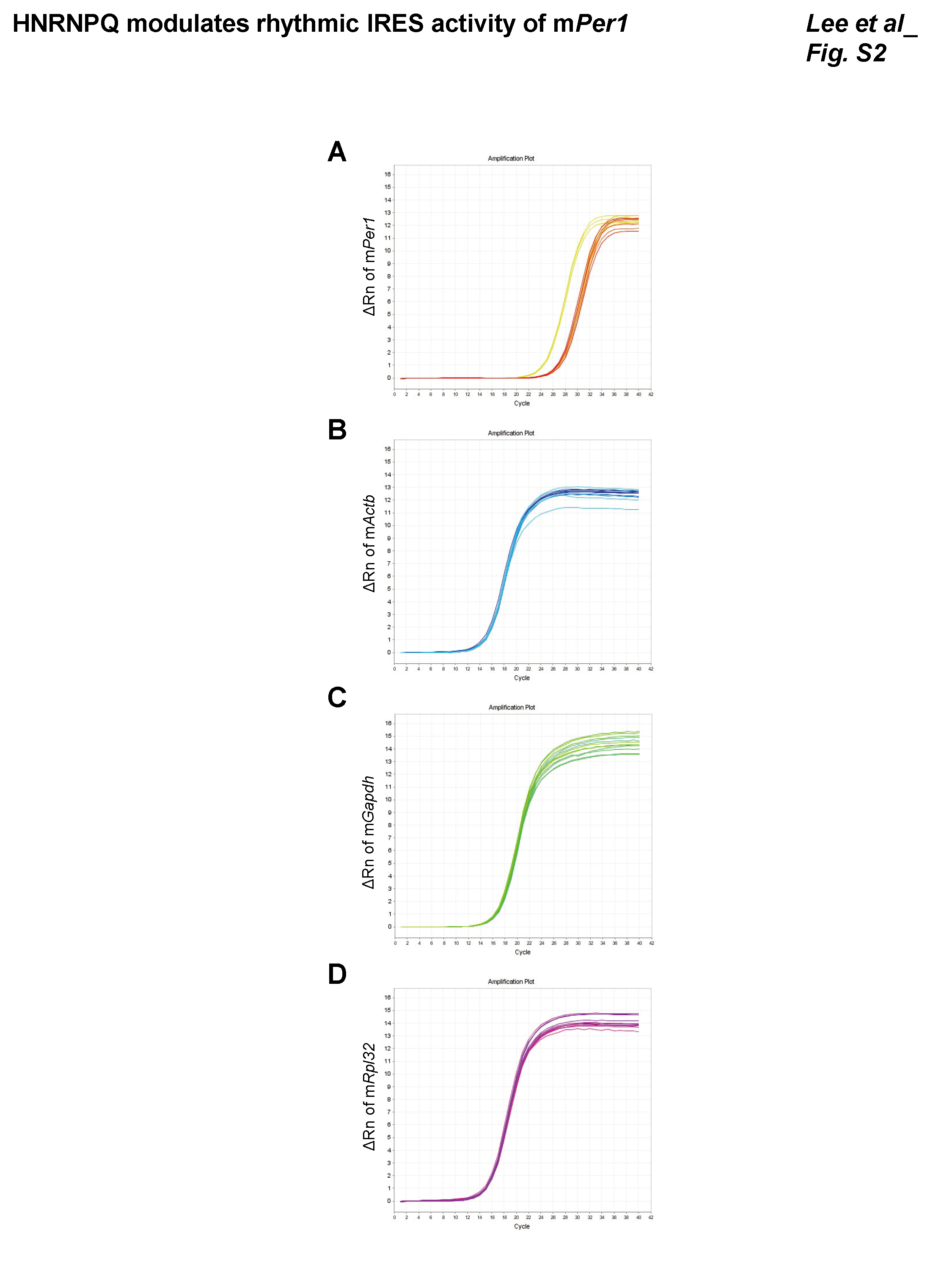

Supplement: Figure S2 — Amplification plots of real-time PCR. Vehicle (DMSO)-, rapamycin (Rapa)-, or cycloheximide (CHX)-treated NIH 3T3 cells were harvested at the indicated time points; then mRNA levels were checked by quantitative RT-PCR with specific primers (Figure 1B–E). To indicate whether the PCR signals were in the linear range, amplification plots are shown. (A) mPer1, (B) mActb, (C) mGapdh, (D) mRpl32. (TIF) [file pone.0037936.s002.tif]

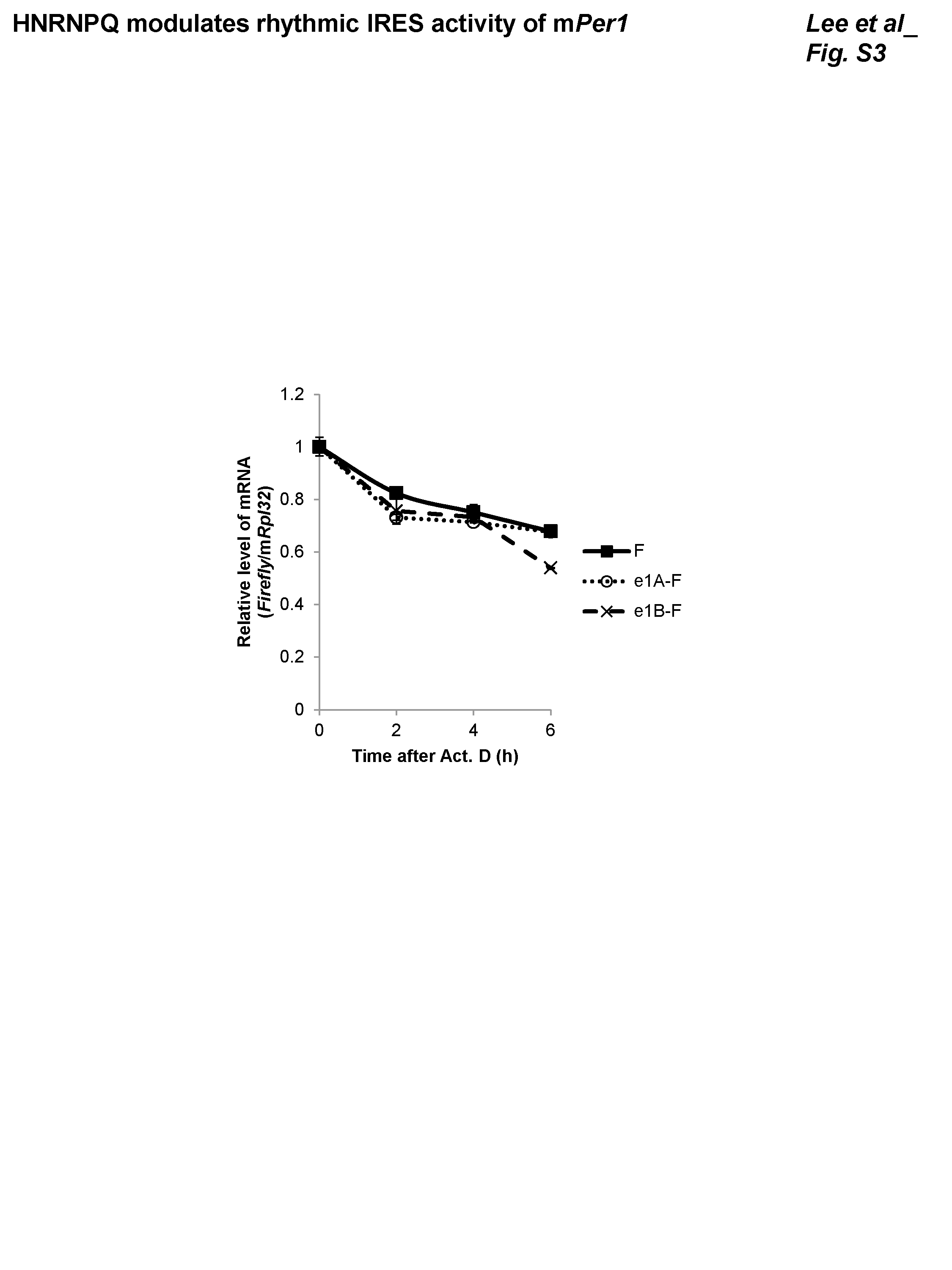

Supplement: Figure S3 — mRNA stability of m Per1 5′UTRs. NIH 3T3 cells were transiently transfected with monocistronic reporter plasmids that 5′UTR is followed by Firefly luciferase. Transfected cells were incubated for 24 h before treatment with 5 µg/ml actinomycin D. Total RNA (1 µg) was reverse transcribed using oligo-dT primer then quantified by real-time PCR. Closed square indicates mRNA levels of Fluc which harbor no 5′UTR. Open circle (e1A) and X (e1B) represent mRNA levels of Fluc which is linked to mPer1 5′UTR. The results are expressed as the mean ± SEM. (TIF) [file pone.0037936.s003.tif]

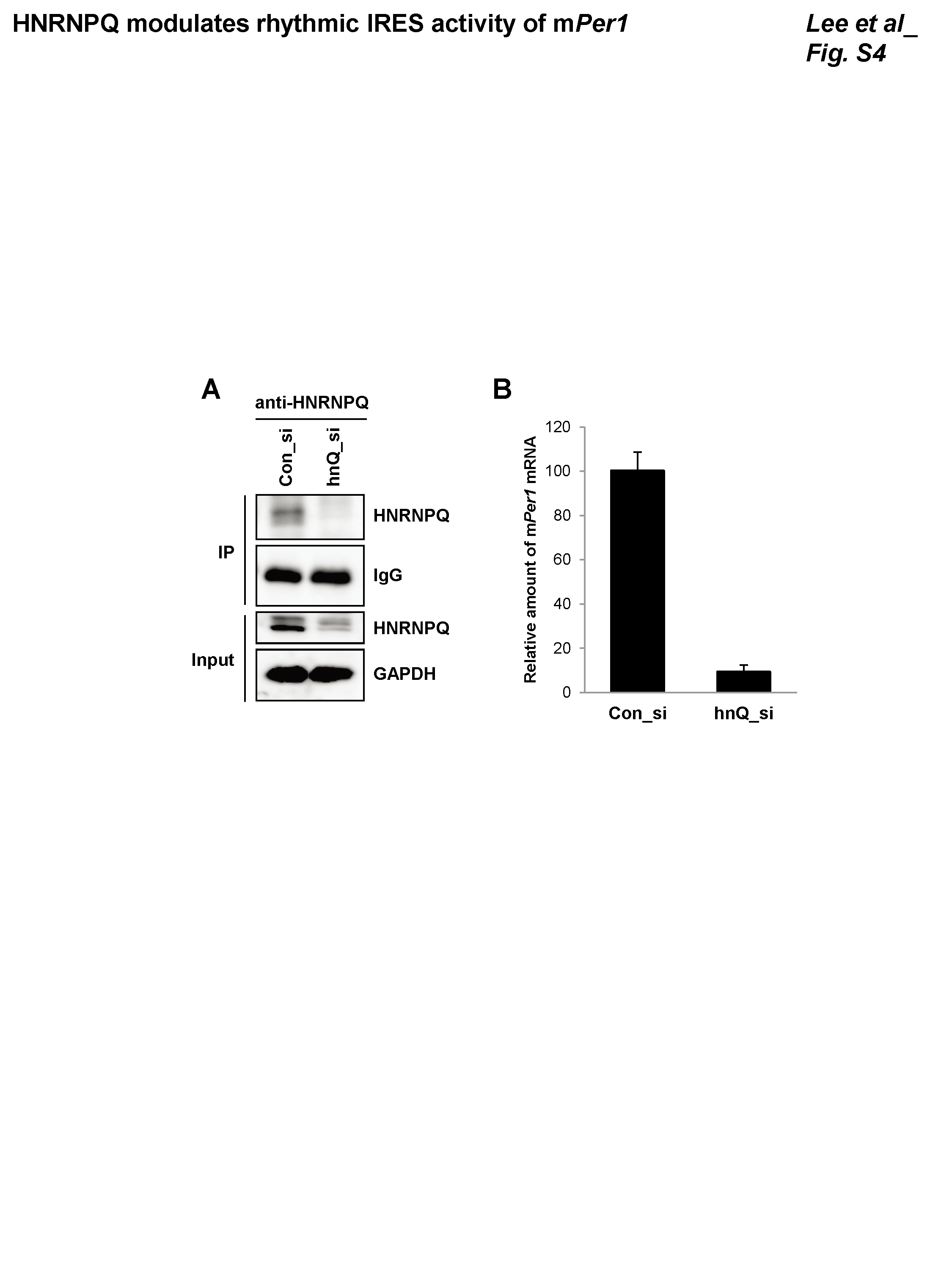

Supplement: Figure S4 — Binding specificity between HNRNPQ and m Per1 mRNA. (A) Cytosolic fraction of NIH 3T3 transfected with Control siRNA (Con_si) or HNRNPQ specific siRNA (hnQ_si) were subjected to IP-RT using HNRNPQ specific antibody followed by immunoblotting. (B) Total RNA was prepared from the one fifth volume of the samples immunoprecipitated with anti-HNRNPQ antibody in panel A, and mPer1 mRNA was detected by real-time PCR. The level of Con_si was set to 100. Error bars represent ±SEM. (TIF) [file pone.0037936.s004.tif]

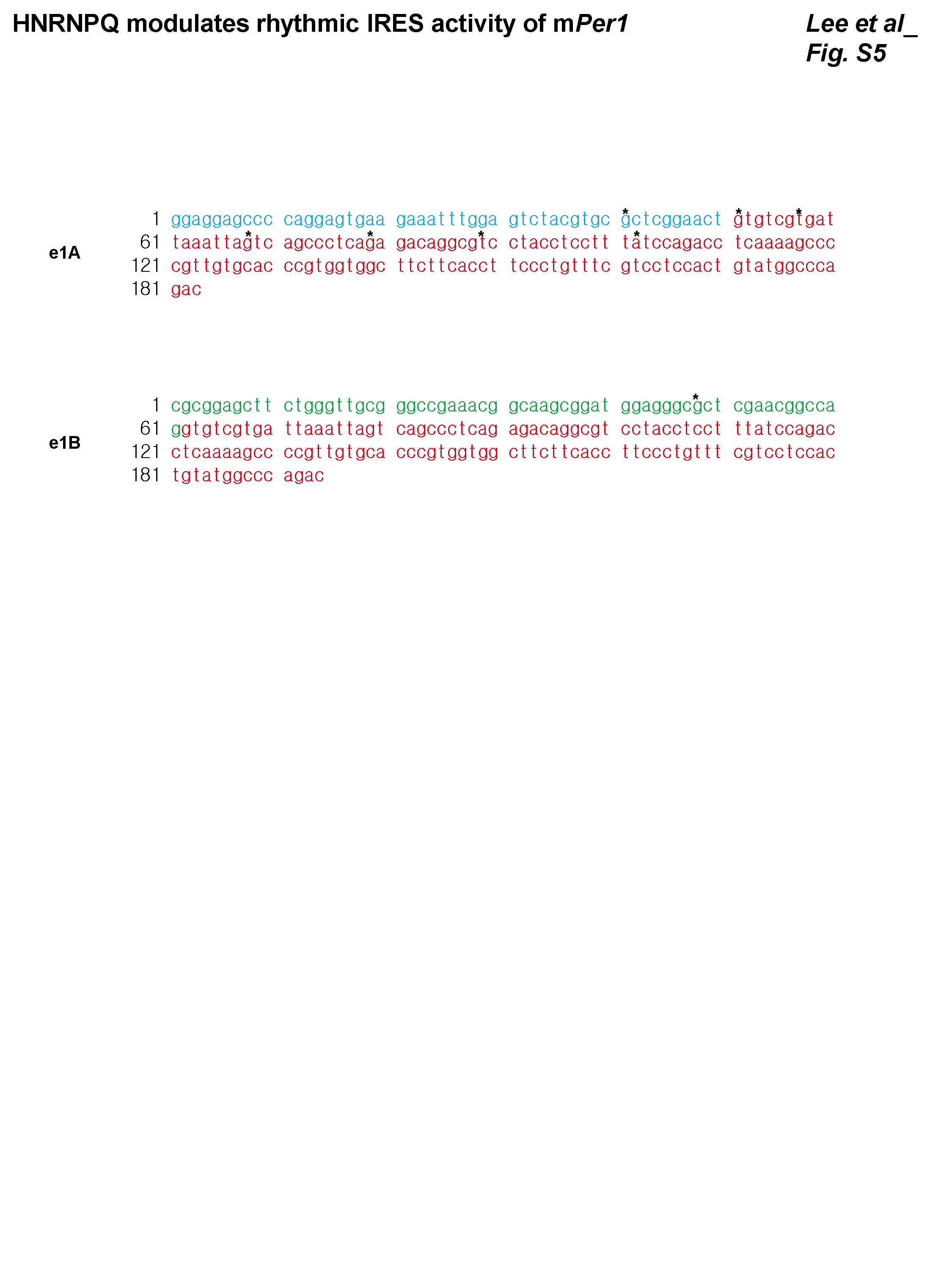

Supplement: Figure S5 — mRNA sequence of the m Per1 5′UTR and the positions of competitive oligonucleotides. 5′UTRs of mPer1, e1A and e1B, were presented. Blue colored sequence is the exon1 of e1A mPer1 5′UTR, green colored sequence indicates the exon1 of e1B. mPer1 5′UTRs e1A and e1B commonly have exon2, which was showed by red color. The starting points of competitive oligonucleotides were depicted as asterisk on the top of nucleotide. (TIF) [file pone.0037936.s005.tif]

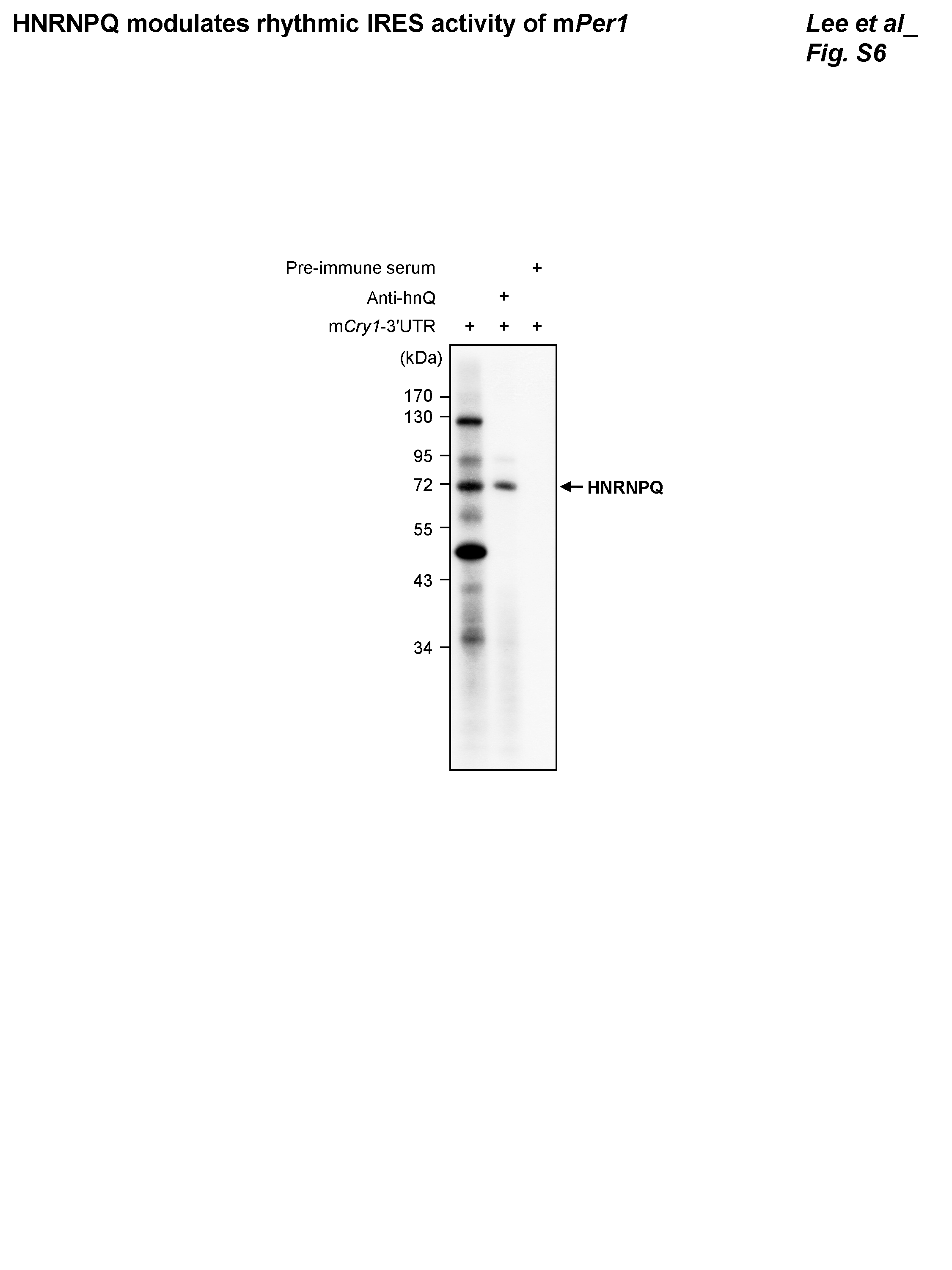

Supplement: Figure S6 — HNRNPQ specifically binds to the m Cry1 3′UTR. 3′UTRs of mCry1 transcribed in vitro were subjected to in vitro binding and UV cross-linking with a cytoplasmic extract. Cytoplasmic extracts labeled by UV cross-linking were subjected to immunoprecipitation with antibodies against HNRNPQ or pre-immune serum as a control. (TIF) [file pone.0037936.s006.tif]

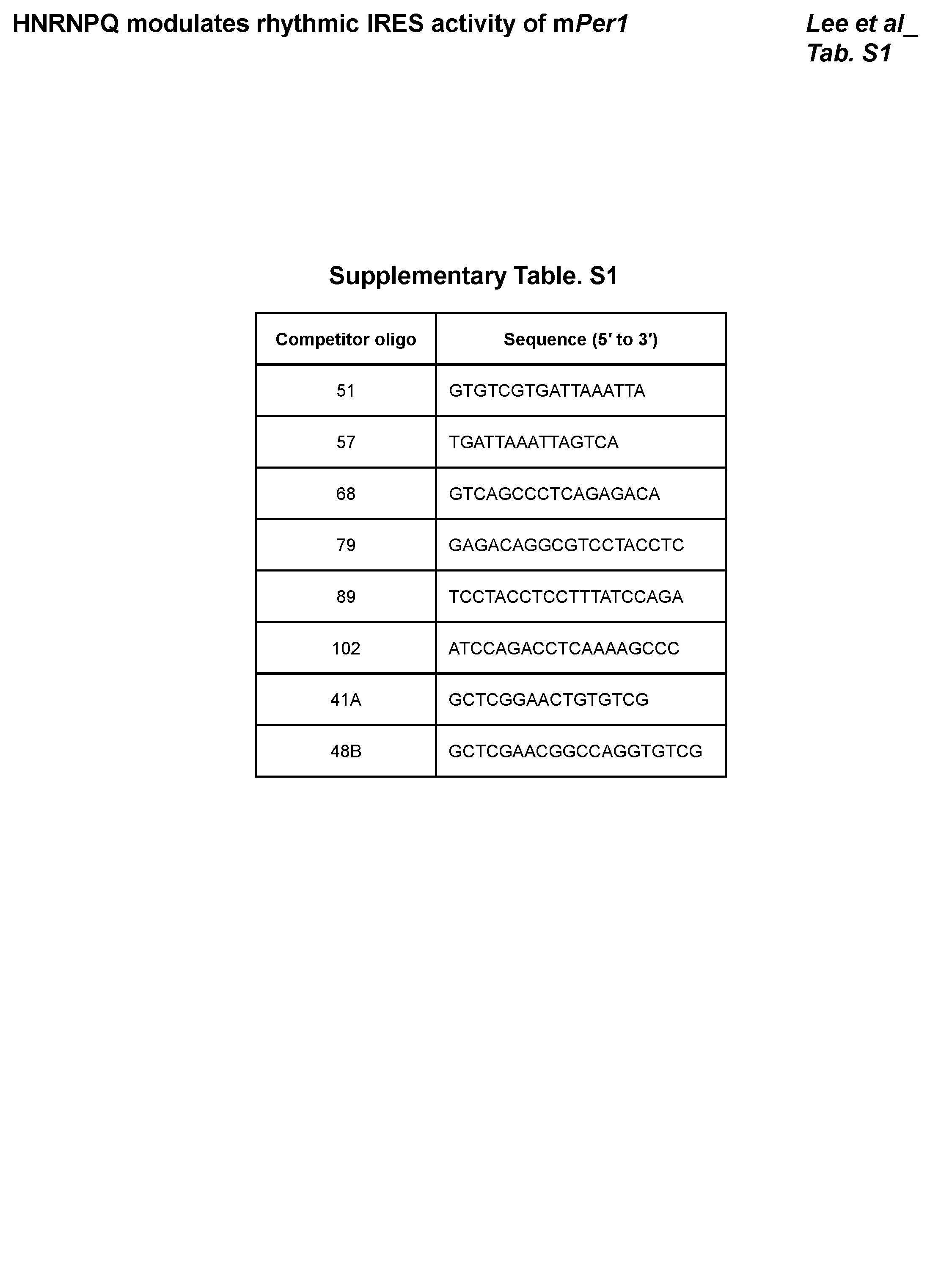

Supplement: Table S1 — Sequences of competitive oligonucleotides. (TIF) [file pone.0037936.s007.tif]
